# Notes and Notices

**Published:** 1883-04

**Authors:** 


### NOTES AND NOTICES.

REMOVED.—Dr. E. W. Berridge has removed from No. 4 Highbury, New Park, to No. 48 Sussex Gardens, Hyde Park, London.

HOMŒOPATHIC MEDICAL SOCIETY OF OHIO.—Next session will meet at Columbus, May 8th and 9th. Dr. H. E. Beebe is still the efficient secretary, and the Society is prosperous.

EXPOSING HOMŒOPATHY.—A Dr. Herrick delivered recently a lecture on Homœopathy at the Cleveland Medical School. He says: "There is but one medical science, and that is in the regular [homœopathic] school. There is no literature in the homœopathic systems." We often wonder why the allopaths find it necessary to lecture so often upon a school that is "*dead.*"

ABOLISH THEM.—The [allopathic] doctors are always discovering some new disease. Let's abolish the doctors.—*Evening Telegraph.* Homœopathic doctors are always discovering some new remedy to cure disease. Let's encourage them.

NO EAR FOR MUSIC.—It is said a medical student declined purchasing Hahnemann's *Organon* because he had no ear for music!

RECOGNITION.—Homœopathy does not need recognition so much at the hands of the allopaths as it does from its own practitioners. When they all recognize and act upon its law there will be no allopaths left to dispute its claims.

FLOATING KIDNEY.—The frequency of this anomaly is greater than is supposed, according to Dr. Skorczewski. Among 1,422 persons examined, he found thirty-five subjects (thirty-two females, three males). In five of these both kidneys were movable. The causes are: pendulous abdomen, disappearance of circumrenal fat, atony of tissues subsequent to febrile diseases, and the pressure of some hypertrophied abdominal viscera. In Dr. Skorczewski's cases there was very often observed coexistence of floating kidneys with malarial hypertrophy of the spleen.—*N. Y. Medical Record.*

